# The threshold force required for femoral impaction grafting in revision hip surgery: A preliminary study in sow femurs

**DOI:** 10.3109/17453674.2010.480936

**Published:** 2010-05-21

**Authors:** Olivia M Flannery, John R Britton, Peter O'Reilly, Nicholas Mahony, Patrick J Prendergast, Paddy J Kenny

**Affiliations:** ^1^Trinity Centre for Bioengineering, School of Engineering, Trinity College, DublinIreland; ^2^Department of Orthopaedics, Cappagh National Orthopaedic Hospital, Finglas, Dublin; Connolly Hospital, Blanchardstown, DublinIreland; ^3^Human Performance Laboratory, Anatomy Department, Trinity College, DublinIreland

## Abstract

**Background and purpose:**

Femoral impaction grafting requires vigorous impaction to obtain adequate stability without risk of fracture, but the force of impaction has not been determined. We determined this threshold force in a preliminary study using animal femurs.

**Methods:**

Adult sow femurs were used because of their morphological similarity to human femurs in revision hip arthroplasty. 35 sow femurs were impacted with morselized bone chips and an increasing force was applied until the femur fractured. This allowed a threshold force to be established. 5 other femurs were impacted to this force and an Exeter stem was cemented into the neomedullary canal. A 28-mm Exeter head was attached and loaded by direct contact with a hydraulic testing machine. Axial cyclic loading was performed and the position sensor of the hydraulic testing machine measured the prosthetic head subsidence.

**Results:**

29 tests were completed successfully. The threshold force was found to be 4 kN. There was no statistically significant correlation between the load at fracture and the cortex-to-canal ratio or the bone mineral density. Following impaction with a maximum force of 4 kN, the average axial subsidence was 0.28 mm.

**Interpretation:**

We achieved a stable construct without fracture. Further studies using human cadaveric femurs should be done to determine the threshold force required for femoral impaction grafting in revision hip surgery.

## Introduction

The use of impaction grafting in total hip arthroplasty was first introduced by Slooff et al. (1984) for restoring bone stock loss in the acetabulum. Small cavities in the acetabulum are first filled, and then the whole socket is filled layer by layer with bone chips impacted with increasing-sized impactors and a hammer. If this procedure is used correctly, a stable layer of graft will result. A modification of the technique for the femoral side was described by Gie et al. (1993). This technique involves adding morselized fresh frozen allograft into the femoral canal and packing the graft tightly into the femoral canal to fill the defects. Impactors, which are attached to a sliding hammer, are used to vigorously impact the bone graft. There are various sizes of distal and proximal impactors. Using these impactors, a neo-medullary canal is created fitting the stem to be inserted.

Evidence of revascularization, retrabeculation, and recorticalization of impacted allograft has been shown using bone scintigraphies (Tokgozoglu et al. 2000) and radiographs (Gie et al. 1993, Linder 2000, van Biezen et al. 2000). Histological studies in animals (Schreurs et al. 1994) and humans (Ling et al. 1993, Ullmark and Linder 1998) have supported radiological studies by showing graft revascularization and remodeling. Ullmark and Linder (1998) showed that revascularization had proceeded to the cement surface in most areas in a femur retrieved 6 months after impaction grafting with morselized allograft.

Results to date are promising, with improvement in hip and pain scores and radiological signs of retrabeculation (Flugsrud et al. 2000, Schreurs et al. 2006). However, there still remains concern about the high rate of complications, especially fractures of the femur. Extramedullary augmentation with a strut graft or plate is used when there is concern regarding large cortical defects. Although their use has been shown to be advantageous, the risk of fracture remains. Furthermore, unacceptably high rates of subsidence have been reported (Eldridge et al. 1997, Masterson et al. 1997), indicating inadequate impaction of the bone chips. Increasing the impaction force will increase graft stability. However, the vigorous process of impaction grafting has been reported to result in femoral fractures with rates as high as 16% (Masterson et al. 1997, Leopold et al. 1999, Knight and Helming 2000, Pekkarinen et al. 2000). This is perhaps because the bone graft is impacted manually with a sliding hammer without any guidance as to the optimal magnitude of the impaction force.

In this preliminary study, we determined the threshold force required for impaction grafting of the femoral component in an animal femur. Factors such as femoral cortical defects, cortex-to-canal diameter, and bone mineral density (BMD), which have been associated with fracture (Ornstein et al. 2002, Meek et al. 2004, Bagger et al. 2006), were studied to determine whether there was any correlation between them and the threshold force. Finally, we measured the subsidence of an Exeter prosthesis under cyclical loading following impaction at the threshold force.

## Materials and methods

After examination of several kinds of animal femurs, adult sow femurs were selected. The femurs were sealed in plastic and stored in a freezer at –20ºC. Before testing, they were thawed in a refrigerator at 4ºC for 24 h. The femurs were stripped of soft tissue and the heads were excised and the neck trimmed, as for a hip replacement. The femoral canal was cleared, leaving only the cortical shell, and the condyles excised. The Gruen zones were marked on each femur before being placed through a DEXA scanner. Each femur was scanned twice and the average BMD overall and for each Gruen zone was determined.

The distal part of the femur was held upright in a cement base. Strain gauges were applied as outlined by Dabestani (1992) to the medial, lateral, anterior, and posterior aspects of the proximal femur at the level of the base of the lesser trochanter.

**Figure 1.**
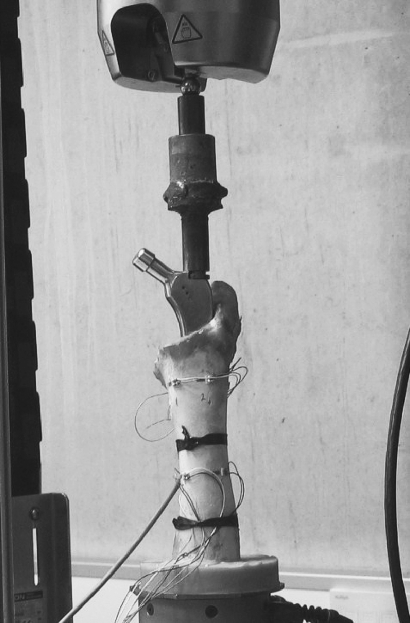
Impaction using the X-Change III proximal impactor 37.5 (2) attached to a biaxial hydraulic fatigue testing machine.

The excised femoral heads and femoral condyles were used to obtain bone grafts. Schreurs et al. (2001) recommended that the size of bone chips should be 7–10 mm on the acetabular side and we followed this recommendation in our experiment, so a rongeur with optimal bite of 10 mm was used. The bone chips were rinsed in tepid water to remove excess fat and patted dry with tissue paper. Bone chips were inserted into the femoral canal and using the X-Change III distal impactor (Stryker Howmedica, Berkshire, UK), the chips were compacted manually until the graft level was approximately 10 cm from the tip of the greater trochanter. The X-Change III proximal impactor (Stryker Howmedica, Berkshire, UK) was then attached to a biaxial hydraulic fatigue testing machine (Instron Inc., Norwood, MA) (Figure 1) and driven into the bone chips at an increasing force.

A force was gradually applied in the Instron until 1.5 kN was reached. An impact force was then applied using the hydraulic testing machine by first setting the machine at a frequency of 5 Hz with Haversian square wave form. An increasing force of 0.5 kN was then applied and recorded until the femur fractured or the second mark on the impactor was in line with the cut of the neck. If the femur did not fracture then, more bone chips were added and the impaction process was repeated until the femur fractured. The graft in the femoral canal was then inspected and the level was noted to be distal or proximal to the base of the lesser trochanter, where the strain gauges had been applied. The strain gauges gave measurements for the strain at the bone surface during impaction. Using callipers, the cortical thickness of the bone was measured along the fracture line.

**Figure 2.**
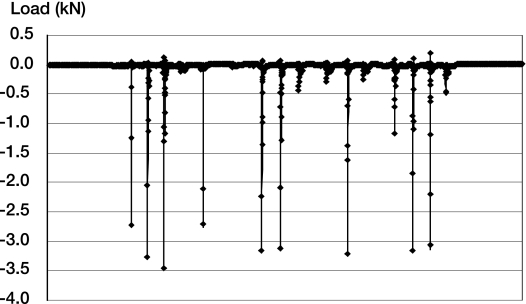
The X-Change III slap hammer used in revision hip arthroplasty was impacted against the load cell. The maximum load obtained was 3.5 kN.

To estimate the force applied in the clinical setting, the Exeter slap hammer was placed directly on the load cell of the biaxial hydraulic fatigue testing machine and the maximum load obtained from manual use by the authors was noted (Figure 2).

**Figure 3.**
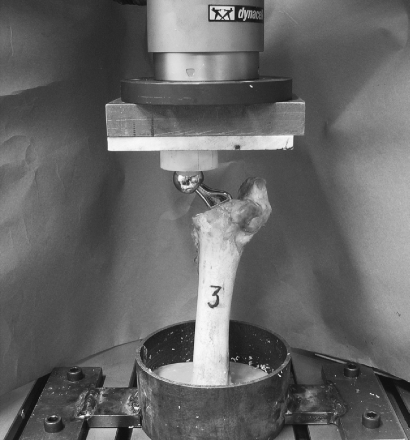
Axial cyclic loading between 440 N (swing phase of gait) and 1,320 N (stance phase of gait) through the 28-mm femoral head, by direct contact with the hydraulic fatigue testing machine via a PTFE disc, which was free to slide along a PTFE plate.

Once the threshold force was determined, 5 femurs were impacted to the threshold force and an Exeter 37.5-mm offset size-1 stem was cemented into the neomedullary canal and a 28-mm Exeter head attached. Each femur was cemented at its base with an abduction angle of 7 degrees and a flexion angle of 15 degrees, which is the natural alignment of the proximal human femur. The femur was then loaded through the 28-mm femoral head by direct contact with the hydraulic fatigue testing machine (Figure 3).

Axial cyclic loading was performed between 440 N (swing phase of gait) and 1,320 N (stance phase of gait) (Malkani et al. 1996, Kligman et al. 2003) for 150 × 10^3^ cycles at a frequency of 3 Hz. The position sensor of the hydraulic testing machine measured the axial displacement of the femoral head. The initial loading of the implant to 880 N was not recorded. Subsidence was defined as the difference between the first and last cycle under 440 N load.

### Statistics

Regression techniques were applied to determine whether a correlation existed between the force applied and calculated bone surface strain levels, cortical thickness, canal diameter, and bone density.

## Results

35 femurs were marked, scanned, and tested. For 2 femurs, the strain gauges failed during testing and they were excluded. A fracture occurred on removing the proximal impactor in 3 femurs and a fracture was noted at the early stages of testing in another femur before impaction grafting was started. As a result, the testing was complete and successful for 29 femurs (Table).

**Table:** Characteristics of the 29 femurs

Test	Load at # (kN)	Strain at # (istrain)	Cortical thickness at # (mm)	Cortical thickness: canal diameter	BMD (g.cm^²^)
1	7.5	6050	3.7	0.14	1.02
2	7.5	4330	5.0	0.21	1.11
3	6.5	2030	4.1	0.15	1.41
4	9	15800	3.7	0.14	1.04
5	8.5	2670	3.6	0.15	1.03
6	4	2250	3.6	0.18	1.15
7	6	6500	3.3	0.15	1.09
8	10	9190	3.4	0.14	1.03
9	7	1870	3.0	0.13	0.95
10	8	5190	3.7	0.15	0.96
11	9.5	4620	3.5	0.14	0.93
12	5.5	1820	3.3	0.14	1.02
13	8.5	3300	5.3	0.22	1.26
14	6	5950	3.6	0.16	1.05
15	8.5	1810	3.2	0.12	1.26
16	5.5	7330	3.0	0.13	1.12
17	9.5	5960	4.6	0.19	1.35
18	6.5	5120	3.6	0.20	1.04
19	6.5	6900	3.6	0.15	1.39
20	8	2700	3.8	0.17	1.40
21	6	4580	4.1	0.16	1.33
22	5	1040	4.9	0.20	1.11
23	8.5	4390	4.3	0.15	1.04
24	8.5	5030	4.4	0.18	1.22
25	10	4100	3.9	0.16	1.25
26	8	8940	4.3	0.18	1.06
27	9.5	3910	4.0	0.15	1.17
28	10	11000	3.6	0.14	1.29
29	10	2660	4.0	0.18	1.20

The threshold force was found to be 4 kN, i.e. all femurs fractured at a greater load. This threshold force was similar to the maximum force obtained when the slap hammer was impacted on the load cell directly, which was 3.5 kN (Figure 2).

The bone surface strain at failure ranged between 1,040 and 15,800 microstrain. There was no correlation between the load applied and the resulting strain. In 20 femurs, where the level of the bone graft was proximal to the base of the lesser trochanter, the average strain at fracture was 6,246 microstrain. For the remaining 9 femurs where the bone graft was distal to the lesser trochanter, the average strain at fracture was 2,459 microstrain. The maximum strain at failure was noted from the strain gauge positioned medially in 19 femurs and from the lateral strain gauge for the remaining 10 femurs. The position of the fracture varied, but most often occurred on the medial aspect of the proximal femur. In 19 femurs, the maximum strain was recorded from the strain gauge at or closest to the fracture.

The average cortical thickness at the level of the strain gauge where the fracture occurred was 3.9 (3–5.3) mm. The mean canal diameter was 24 (18–28) mm and the mean cortex-to-canal ratio was 0.16 (0.12–0.22). There was no statistically significant correlation between the load at fracture and these parameters.

The average BMD was 1.16 (0.92–1.44) g/cm^2^. However, there was a slight variation in the length of the femurs so the average BMD was calculated for the femoral length excluding Gruen zone 4. This average BMD was 1.22 (0.96–1.5) g/cm^2^. There was no statistically significant correlation between the average BMD and the load of fracture or between the BMD for each Gruen zone and the load (r^2^ = 0.0031). An r^2^ of > 0.7 is required for the correlation to be statistically significant.

**Figure 4.**
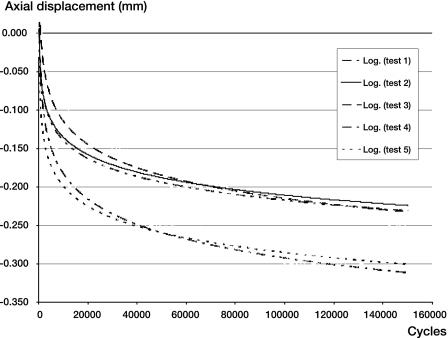
Axial displacement of implant during swing phase of gait for each femur.

Following impaction with the maximum force of 4 kN, the average subsidence for the 5 femurs after 150 × 10^3^ cycles at a frequency of 3 Hz was 0.28 (0.24–0.33) mm (Figure 4).

## Discussion

The high risk of fracture during femoral impaction grafting is of some concern. Knight and Helming (2000) reported a fracture rate of 16% related to the impaction grafting technique. Similar figures have been reported by Leopold et al. (1999) and by Pekkarinen et al. (2000).

Our experimental animal study was designed to determine the threshold force until fracture for femoral impaction grafting. We selected adult sow femurs as they lack trabecular bone between the lesser trochanter and femoral condyles, and have a wide femoral canal with a thin cortex (Höstner et al. 2001). These characteristics mimic the human femur in revision hip arthroplasty. Femurs were thawed in a refrigerator at 4ºC for 24 h before testing. The mechanical properties and biochemical behavior of dead and living bone are similar and the mechanical properties of bone are not affected by freezing (Dabestani 1992). However, one of the limitations of our study is that the full length of the femur, after excision of the condyles, was used. This meant that the overall shape was not only curved but more cylindrical than conical. The femoral canals were also wide, with an average diameter of 24 mm. The diameter in the clinical situation is up to 16 or 18 mm. This large diameter of the canal explains why we could use chips with a diameter of 7–10 mm; in the clinical situation these chips are too large to be used for distal impaction. Although this resulted in a very thick bone graft mantle using the Exeter 37.5-mm offset size-1 stem, larger stems could not be used because they did not fit the sow femur proximally.

The thinnest part of the cortex for the femurs tested was 3.2 mm on average, but there were no cortical defects. In revision hip surgery, large cortical defects of the femur can be encountered. Thus, although the threshold force of 4 kN is similar to that obtained from direct impaction with the Exeter slap hammer on the load cell (3.5 kN), this is unlikely to be obtained in the clinical situation—and if it was reached, the risk of fracture would probably be high. In revision surgery, cages, meshes, cerclage wires, or strut grafts are used to reinforce a thin or absent cortex. This was not considered in our animal study, as no cortical defects were encountered.

Another limitation of our study is that the bone had to be stripped of soft tissue and was fixed and potted in cement, resulting in an aphysiological set-up. During revision hip arthroplasty, the femur is embedded in soft tissues and is not in a fixed stable position. Therefore, the forces obtained in our animal study may never be reached clinically. Thus, the methods we used are inappropriate in some respects but we attempted to put some boundaries on the forces applied in femoral impaction grafting to reduce the risk of fracture.

We expected the bone mineral density, cortical thickness, and canal diameter to be correlated to the load but this was not found. The reason for this is that failure most likely occurs from some isolated defect in the bone cortex rather than being due to a low overall density. We used callipers to measure the cortical thickness along the fracture line. For femurs where the maximum resultant strain was not at the fracture site, the cortex was cut and the thickness measured. The canal diameter was measured from the images obtained from the DEXA scan, and in retrospect plain radiographs should have been used to obtain more accurate measurements of both cortical thickness and canal diameter.

The initial stability is of paramount importance in femoral impaction grafting. Inadequate impaction of the bone chips is a possible reason for early massive subsidence (Eldridge et al. 1997). The mechanical stability is related to the compaction of the graft (Toms et al. 2004) but over-impaction can result in an intraoperative fracture. Minimal axial subsidence occurred in the 5 femurs tested, where the mean subsidence was 0.28 mm. This was after 150 × 10^3^ cycles at a frequency of 3 Hz, which would simulate the first 2 months of load bearing.

In conclusion, a threshold force of 4 kN was determined in this preliminary study. Minimal axial subsidence of the implant occurred when impacting the graft with this threshold force. We therefore achieved a stable construct without fracture. We have started further studies in human cadaveric femurs using radiographs to measure the canal diameter and cortical thickness, in order to determine the threshold force required in femoral impaction grafting in revision hip surgery.
